# Migration Versus Immobility, and Ties to Parents

**DOI:** 10.1007/s10680-018-9494-0

**Published:** 2018-08-07

**Authors:** John Ermisch, Clara H. Mulder

**Affiliations:** 1 Department of Sociology and Nuffield College, University of Oxford, Manor Road, Oxford, OX1 3UQ UK; 2 Population Research Centre, Faculty of Spatial Sciences, University of Groningen, P.O. Box 800, 9700 AV Groningen, The Netherlands

**Keywords:** Migration, Local ties, Family ties, Parent–child contact, Geographic proximity

## Abstract

We investigate the association between geographic proximity to parents and the likelihood of moving longer distances (e.g. at least 40 km), using British panel data from the Understanding Society study and probit regression. We also look at the extent to which this association diminishes by introducing measures of frequency of contact, interaction with neighbors and length of residence. Using a number of different models and samples, we find that living far from parents increases longer distance mobility. Seeing parents weekly and more interactions with neighbors reduce longer distance mobility, but its association with parental proximity remains substantial. The positive effect of living far from parents on the likelihood of moving longer distances is also found in subsamples of those who have lived in their current residence for 5 years or less and of the highly educated, while the negative effect of seeing parents weekly is also found in these subsamples as well as in a subsample of those living close to parents. Even though endogeneity cannot be ruled out completely, these findings show a robust association between family ties and the likelihood of moving a long distance.

## Introduction

Migration is an important way for people to improve their position in the labor market. At the same time, migration leads to severing ties to local social networks, including those to family. As distance is a strong predictor of contact and support exchange between family members (Lawton et al. 1994; Joseph and Hallman 1998; Hank 2007; Bordone 2009; Hank and Buber 2009; Mulder and Van der Meer 2009), migration away from family will almost certainly be associated with a decrease in contact and support. Because family members, and particularly adult children, are often of major importance in the parents’ social networks (Herbers and Meijering 2015) and the main providers of support (Bengtson 2001; Komter and Vollebergh 2002; Spitze and Logan 1990), migration away from parents might have severe consequences for the parents’ contact and support networks. It is therefore valuable to study migration in relation to local ties to parents.

Previous migration research has rarely taken local ties to parents into account, but there are some exceptions. In Sweden, a remarkably strong negative association was found between having parents or siblings living close and couples’ and families’ likelihood of migrating (Mulder and Malmberg 2014). Michielin et al. (2008) found that having parents living nearby reduced the likelihood of migrating. A negative association was also found between having parents or siblings living close and moving at the occasion of separation (Mulder and Malmberg 2011, for Sweden; Mulder and Wagner 2012, for the Netherlands). Family living nearby was negatively associated with migration intentions and actual migration of young people in two German cities (Vidal and Kley 2010). In the USA, low-income households have been found to move less frequently out of their neighborhood if they had relatives living in that neighborhood (Dawkins 2006; see also Spilimbergo and Ubeda 2004).

The previous studies only provided evidence of an association between family or other network members living close by and the likelihood of migrating or moving from the neighborhood. It is not clear from these studies whether actual contact or support exchange with family prevented people from migrating, or whether there was some other mechanism underlying this association—for example, knowledge of the local labor market or having ‘weak ties’ to people nearby obtained through the parents. The only research we know of that investigated the impact of actual social ties on migration was Belot and Ermisch’s (2009) study on the impact of friendship ties on the likelihood of geographic mobility. Their findings indicated that a larger number of intimate friends living in the same neighborhood had a substantial negative effect on the probability of moving 20 km or farther.

In this study, we extend the literature on the association between having parents living close by and the likelihood of migrating in two ways. Firstly, we study this association in the context of Britain, which is generally seen as a liberal welfare state that provides only limited state support. Secondly, we go beyond investigating the mere association between living far from or close to parents and migrating by also looking at how actual contact with family members, interactions with neighbors and duration of residence are related to this association. We use the first four waves of the Understanding Society survey for Britain to estimate probit regression models of moving longer distances, varying the ‘distant’ cutoff from at least 10 km to at least 50 km.

## Theoretical Framework

### Ties to Parents

Our theoretical framework is based on the transaction costs approach pioneered by Weinberg et al. (1981) and Venti and Wise (1984). From this perspective, changes in desired residential location, triggered by job, housing or other reasons, give rise to disequilibrium in people’s location, and adjustments in response require moving house. Because moving is a costly process, moves are only made when the benefits from the desired location deviate from those at the present one by a sufficiently large amount, with the threshold being a function of the costs of moving. These transaction costs may be financial or social. The contribution of social ties outside the household to these costs of mobility has long been recognized (e.g. McGinnis 1968). More recently, the term ‘local social capital’ (Kan 2007) has been used to refer to household resources that arise from social ties or networks, for example the number of close friends living locally (Belot and Ermisch 2009), having someone nearby to turn to in an emergency (Kan 2007) or contact with neighbors and family (David et al. 2010). The existence of such local networks has been found to deter moves, especially longer distance moves (Kan 2007; Belot and Ermisch 2009; David et al. 2010; Mulder and Malmberg 2014).

Parents are usually important in most people’s social networks as valuable sources—and also recipients—of social contact, emotional closeness, various kinds of support and information; geographic proximity facilitates interaction; and, therefore, parents form a strong local tie. Our point of departure is a simple main hypothesis: That the local presence of parents keeps people from migrating, and thus, that those whose parents live far away are more likely to migrate (i.e. move longer distances) than those whose parents live close by (compare Mulder and Malmberg 2014).

A statistical association between distance to parents and the likelihood of migrating need not be evidence of a substantive association. Rather, the association could be (partly) spurious and caused by other ties to the locale, and it is therefore important to account for such other ties and to measure better the parental ties. We introduce one measure of parental ties—frequency of physical contact with them. It is also important to acknowledge possible endogeneity between contact, proximity to parents and migration trajectories. For example, there may be people who dislike change and therefore stick to parents for contact and to a familiar environment for their residential location, which in turn may strengthen other local ties, such as with neighbors.

Furthermore, it should be acknowledged that the parents might move rather than the adult children and that some of the moves of adult children who live far from parents might be directed toward the parents—for example, if they are undertaken to facilitate support exchange or other forms of contact with parents.

### Other Local Ties

The expected costs of moving to a new location, and their relative weights in the decision to move, vary according to household circumstances. We focus on ties to parents, but also incorporate other circumstances identified from previous research in Britain and elsewhere to be strongly associated with both mobility and the level of financial or social costs of mobility: having children, neighborhood ties, housing tenure, having a partner and duration of residence.

Many previous studies of residential movement claim that the numbers of children at different ages affect returns to or costs of movement (e.g. Fischer and Malmberg 2001). The social costs of a move tend to be higher among individuals with children than among those without children. Social costs for those with children include the upheaval of changes in school and childcare arrangements and disruption of their children’s social networks. In Britain, those with children also report having more friends living nearby than their childless counterparts (Belot and Ermisch 2009), while in the USA those with children are more likely to have someone close by to call upon in an emergency (Kan 2007). Furthermore, loss of ties to neighbors is another potential cost of mobility, particularly longer distance moves.

Selling fees and stamp duty land tax raise the transaction cost of a move for owner–occupiers relative to renters, while the social costs may be higher for homeowners because of a greater investment in forming local ties (DiPasquale and Glaeser 1999; Kan 2007). British social tenants (renting from a local authority or housing association) face higher transactions costs (albeit different in nature) than private tenants, with local authority tenants finding it particularly difficult to move across local authority boundaries (Boyle and Shen 1997; Thomas et al. 2015). According to the family migration literature (summarized by Cooke 2008), people who have a live-in partner will find it more difficult to make longer distance moves because it requires agreement between the partners.

Next to ties to parents, children, neighborhoods, homes and partners, there are likely to be other local ties (e.g. knowledge of the local labor or housing market). These ties strengthen with the passage of time (duration of residence), which is sometimes called ‘cumulative inertia’ (McGinnis 1968). Gordon and Molho (1995) presented a theory in which individual evaluations of the relative value of alternative locations are assumed to evolve stochastically, reflecting changes in individual circumstances that reduce satisfaction with the current residence (‘cumulative stress’). When combined with cumulative inertia, a non-monotonic relationship between the probability of moving and duration of residence at the current address emerges, with probabilities of movement starting at 0, rising quickly over the first few years and then falling. A variant on this theme, but stressing the effect of movement rather than immobility, is that experience of movement ‘may foster a learning process that blunts subsequent inertia’ (Morrison 1971), so that people with more experience of movement are overrepresented at shorter durations of residence. Of course, heterogeneity in mobility propensities also overrepresents less mobile people at longer durations.

### The Benefits of Moving and Demographic Factors

The focus of the paper is on mobility costs, particularly local family and social ties, but it is also important to take into account what may make people desire to move a longer distance. Job opportunities are an important reason that people move longer distances. Better educated people are likely to face a distribution of earning opportunities that has a larger variance, making them choosier in the jobs that they accept and causing them to search longer and over a wider geographic area. Job opportunities requiring a higher level of education may also be more dispersed geographically. The higher income and greater wealth of the better educated could also lead them to search for housing opportunities over a broader area. It is therefore no surprise that level of education is an important predictor of the likelihood of migrating (e.g. Fischer and Malmberg 2001). We also expect that people living in rural areas are more likely to move long distances because of lower local density of job and housing opportunities. As a matter of course, we also account for age, gender and whether the respondent was foreign-born.

## Data and Measurement

The data come from a very large national representative household survey from the UK: *Understanding Society* (also known as UKHLS). The first annual wave was collected in 2009–2010 using stratified sampling along the same lines as the *British Household Panel Survey* (BHPS, of which it was the successor) and the *Panel Study of Income Dynamics* (PSID), but also including an ethnic minority boost. There are now seven annual waves (each collected over a 2-year period), but because not all of our key data are collected in every wave, we use the first four waves of data. Each person aged 16 or older answers the individual adult interview and self-completion questionnaires, which ask questions related to local ties, particularly proximity to and contact with parents and interactions with neighbors, and to other variables affecting geographic mobility. Just over 50,000 individuals in about 30,000 households contributed productive interviews in the first wave of the study, including an ethnic minority booster sample of 4000 households. We focus on people with a living parent who does not co-reside with them (35,721 person-year observations).

As in all panel surveys, there is wave-on-wave dropout (and re-joiners), and dropout is likely to be higher among people who move house. Among the general population sample members (as distinct from the ethnic minority booster sample) who completed the individual interview at wave one—and excluding those known to have died by the time of wave two—75.4% were interviewed again at wave two, and proxy interviews were conducted on behalf of a further 1.9% (Lynn et al. 2012). The main issue here is not the extent of attrition, but whether this attrition is ‘ignorable’ for the analysis of the impacts of covariates on residential mobility. ‘Non-ignorable dropout’ arises when residential mobility at each wave is associated with dropout, even after conditioning on other variables. As Washbrook et al. (2014) point out, ‘The problem is particularly relevant to residential mobility because it is plausible to believe that the act of moving house has a direct, or even causal, effect on dropping out.’ In their analysis of BHPS data, the collection of which has very similar tracking and follow-up procedures to *Understanding Society*, Washbrook et al. (2014) find that their substantive conclusions about the impact of covariates on mobility are not strongly affected by assuming that dropout is independent of mobility. The Appendix discusses dropout in more detail, including estimation of the effects of key covariates on it. Overall, we conclude that our parameter estimates may understate the magnitude of the true effects of our covariates on long-distance mobility.

All explanatory variables are measured in the year preceding the potential move. Descriptive statistics of the variables used in the models (based on the final analytical sample) are in Table 1.

**Table 1:** Means and standard deviations of variables in estimation sample

	Mean (SD)
Any move	0.0919
Move at least 40 km	0.0114
Parent lives 1 h or more away	0.36
See parent weekly	0.50
Dependent child	0.50
Neighborhood interaction	− 0.06 (1.00)
Housing tenure	
Private tenant	0.18
Homeowner	0.67
Has partner	0.74
Years in current residence	8.8 (8.7)
Education	
Post-compulsory	0.34
Degree or higher	0.32
Rural	0.21
Age (years)	41.2 (11.1)
Female	0.59
Not UK born	0.16

*N *= 27,043

### The Mobility Measure

Questions relevant to family and neighborhood ties were asked at the first (2009–2010) and third (2011–2012) waves of the study, enabling us to study movement between waves one and two and between waves three and four in relation to these variables. We are particularly interested in residential mobility that affects local social and family networks. Local moves may allow people to maintain their local ties while changing residence, whereas moves that take children away from a close traveling distance to parents can disrupt these networks. Our paper therefore focuses on more distant moves. Of course, where we draw the line between ‘local’ and ‘distant’ moves is somewhat arbitrary and would depend on local topography and transport infrastructure. We focus on moving 40 km or farther, but also examine whether our results change with other cutoff distances. Furthermore, even though we prefer to use the distance threshold approach, we also analyze moving distances conditional on moving as one of our robustness checks.

In the *Understanding Society* data, the household grid indicates whether a person moved address since the last wave, and there are variables (*plnowy4* and *plnowym*) which indicate the year and month in which they moved if they were interviewed at the prior wave and moved address since the last wave. In addition, a variable is created from the postcodes of the addresses before and after the move which indicates how far the people moved (*movdist(wave)* in the XWAVEDAT record). These measures are combined to obtain our ‘distant move’ variable.

### Ties to Parents

#### Proximity to Parents

The family networks module of *Understanding Society* contains a question about which of the respondent’s non-coresident relatives are ‘alive at the moment.’ Those with a mother (father) living outside the household are asked ‘About how long would it take you to get to where your mother (father) lives? Think of the time it usually takes door to door.’ Among those with both parents alive, most are in the same proximity category, in large part because the parents live together. Where the two parents are not living together and are in different proximity categories, we focus on the parent who lives closer.

#### Frequency of Contact with Parents

The family networks module of *Understanding Society* also has information about frequency of contact with parents, which allows us to explore whether people living near their parents behave differently in terms of physical contact with them and whether the much lower mobility rate among those living near reflects such behavior. It contains the following questions: ‘Thinking about your mother (father). Please look at this card and tell me how often you see your mother (father).’ The categories for frequency of seeing parents and traveling time to parents used in *Understanding Society* are discussed in section ‘Exploratory analyses.’

### Other Local Ties

*Dependent children* was measured using a dummy variable for whether any children aged 15 or under were living in the household.

In the self-completion questionnaire of waves one and three, there are a series of statements about the respondent’s *neighborhood*. Respondents are asked to tick a box indicating how much they agree with each statement, ranging from ‘strongly disagree’ to ‘strongly agree.’ Five of these statements are used to construct an index of interaction with neighbors: (1) I feel like I belong to this neighborhood. (2) The friendships and associations I have with other people in my neighborhood mean a lot to me. (3) If I needed advice about something I could go to someone in my neighborhood. (4) I borrow things and exchange favors with my neighbors. (5) I regularly stop and talk with people in my neighborhood. Because of the categorical nature of these responses, multiple correspondence analysis (MCA) was used to calculate an index for neighbor interactions from responses to these five statements (the score for the first dimension employing the MCA analysis).^1^ Its score was coded so that a higher value indicates more interaction. By construction, its distribution has mean zero and unit variance.

*Housing tenure* was measured as a categorical variable with categories social tenant (reference), private tenant and homeowner. We included a dummy for whether a partner was living in the household.

We include a *residential duration* variable in the analysis, based on a question about years residing in the home asked at the first wave and an update in the third wave based on their movement history subsequent to the first wave. Given our dependent variable—the probability of distant moves—a better measure would be duration of living in the community, but we do not have such information.

### The Benefits of Moving and Demographic Variables

*Level of education* was measured as: basic compulsory education or lower (reference category); some level of tertiary education below degree level, and degree or higher. A residence was coded as *rural* if the address falls within urban settlements with a population of less than 10,000.^2^ We also include *age* in years, a dummy for whether the respondent is *female* and a dummy for whether the respondent was *born outside the UK*.

The control variables may not only affect the current chances of moving, but also proximity to parents. For example, geographic mobility tends to move the two generations farther apart (Rogerson et al. 1993), and because the effect of successive residential moves accumulate over the life course, the physical distance between parents and children increases with age (e.g., see Chan and Ermisch 2015). In our data, we find that the following people are more likely to live ‘far’ from their parents: men, older people, childless, those with a partner, better educated, private tenants, those with weaker neighborhood ties, those with a shorter time in the current residence and, of course, those not born in the UK.

## Exploratory Analyses

In our sample, 9.12% moved (see Table 2; number of moves = 3256) and 1.06% moved at least 40 km since the last annual wave (number of moves = 379). Among all moves, three-fourths are less than 10 km. If, as argued above, local moves allow contact with family to be maintained, we would expect the difference in mobility between those whose parents live ‘far’ and ‘near’ should increase with the cutoff distance for ‘long distance mobility.’ Table 2 therefore also shows the odds ratio of moving associated with having a parent living ‘far’ (at more than 1 h traveling distance) compared to ‘near’ (a justification of this distinction between near and far is provided later in this Section). This odds ratio indeed increases with the cutoff distance: Whereas those living ‘far’ are 2.92 as likely to move for the 10 km cutoff, they are 6.42 as likely to move for the 50 km cutoff.

**Table 2:** Differences in ‘long distance’ annual mobility associated with proximity to parents (% moving and odds ratio): different cutoff distances

Distance: equal to or more than	(1) Percentage moving	(2) Odds ratio, ‘far’ versus ‘near’^a^
50 km	0.98	6.42
40 km	1.06	5.81
30 km	1.23	5.00
20 km	1.50	4.06
10 km	2.23	2.92
Any move	9.12	1.63
Move less than 10 km	6.88	1.30

*N *= 35,721

^a^The odds ratio is the probability of moving ‘a long distance’ for those living ‘far’ from parents divided by the probability of moving ‘a long distance’ for those living ‘near’ to parents. It is calculated from a logistic regression of a long-distance move on the variable indicating ‘living far from parents’

The fact that people living ‘far’ from their parents also have a higher ‘short distance’ (less than 10 km) mobility rate than those living ‘near’ suggests that many of the former are just more mobile, and this may be why they are living far from their parents in the first place. In the regression analysis that follows, we address this to some extent by conditioning on years in current residence and by also estimating an OLS regression model of the distance moved which conditions on having moved.

The odds ratios in Table 2 suggest that, at least as regards the association of mobility with proximity to parents, it does not matter much which cutoff distance is used between 30 and 50 km. There is a trade-off between a cutoff which is more in accordance with the theory (a longer distance) and the precision of the estimates, which is related to the number of moves. Between 50 and 30 km cutoffs, the number of moves increases from 352 to 441, and we have chosen a 40 km cutoff (379 moves) as a compromise. We re-estimate our models below with five different cutoffs (10–50 km).

Although parents can also move,^3^ we expect that longer distance moves disproportionally affect proximity to parents. We do not know about moves of parents, but we do know about changes in proximity between waves one and three for respondents present in both. Panel A of Table 3 shows that 5.7% (that is, 2.9 + 2.8%) changed their proximity to parents in terms of the 1-h threshold, with equal numbers moving closer to and farther from parents.

**Table 3:** Changes in living ‘far’ from parents between waves one and three

		Cell %	Row %	Cell %	Row %	Total (column %)
Far_*t*_		0		1		
*Panel A Entire sample* (*N *= *28,384*)						
Far_*t-2*_ = 0		62.3	95.7	2.8	4.3	65.1
Far_*t-2*_ = 1		2.9	8.3	32.0	91.7	34.9
Total			65.2		34.8	100
*Panel B: People who moved at least 40* *km* (*N *= *202*)						
Far_*t-2*_ = 0		7.9	23.9	25.2	76.1	33.1
Far_*t-2*_ = 1		21.8	32.6	45.0	67.4	66.8
Total			29.7		70.3	100

Among the 202 movers of 40 km or more in the 2 years between waves one and three, 47% change their distance relative to parents, with a slightly smaller proportion moving closer to parents compared to moving farther away. Among the 135 movers at risk of moving closer (i.e. they lived an hour or more away in wave 1), 32.6% do so, and among the 67 movers at risk of moving farther away, 76.1% do so.^4^ The large percentage of the latter who end up living more than an hour away indicates that moves of 40 km or farther are very likely to take people beyond an hour’s journey to their parents. Of course, those originally living less than an hour away are less likely to move at least 40 km in the first place.

Table 4 shows the percentage moving at least 40 km by detailed measurements of proximity to the closest parent, frequency of seeing parents and age of children. The primary distinction with regard to proximity appears to be between those whose closest parent lived within 1 h from them and those whose closest parent lived farther away: Only 0.4% of the ‘near’ group moved (91 moves) compared with 2.3% of the ‘far’ group (288 moves): an apparently strong effect of ‘local family ties.’ When we controlled for other factors influencing mobility in regression analysis similar to that reported below, the three categories nearest to parents had about the same mobility rate—hence, our contrast between ‘within 1 h’ (*near*) and 1 h or farther’ groups (*far*).

**Table 4:** ‘Long distance’ (at least 40 km) movement by proximity to parents, frequency of seeing parents, children’s age and combination children’s age/seeing parents

	Moving at least 40 km			
	Percentage	Number of moves		
*Traveling time to closest parent* (*N *= *35,721*)				
Less than 15 min	0.33	45		
Between 15 and 30 min	0.35	21		
Between 30 min and 1 h	0.73	25		
Between 1 and 2 h	2.58	81		
More than 2 h	2.82	153		
Lives/works abroad (spontaneous)	1.29	54		
*Frequency of seeing parent* (*N *= *35,837*)				
Never	0.79	9		
Less often	1.4	67		
Several times per year	2.1	123		
At least once per month	1.99	116		
At least once per week	0.39	52		
Daily	0.32	15		

	Percentage	Number of moves	% Not see weekly	% See weekly
*Presence of child aged…* (*N *= *35,824*)				
None <= 15	1.41	250	2.29	0.46
0–2	1.00	60	1.79	0.36
3–4	0.75	32	1.40	0.18
5–11	0.65	65	1.09	0.28
12–15	0.52	32	0.77	0.28
Aggregation: <=15	0.73	132	1.24	0.29

Table 4 also shows that having at least weekly contact is associated with a much lower probability of moving at least 40 km. Thus, as a measure of ‘frequent contact’, we take those who see their closest parent *at least weekly* (one-half of the sample), of which 0.4% move at least 40 km compared to 1.8% for those with less frequent contact.

Long-distance movement is most likely for those without dependent children in their household, and the chances of movement decline with the age of a child (also in Table 4). The last two columns of Table 4 distinguish between those seeing and not seeing their parents at least weekly. These figures suggest that children inhibit long-distance movement less if respondents do not see their parents weekly. Among those seeing their parents weekly, there is no significant variation in movement with respect to the age of children (*p* = 0.60).

Table 5 shows that seeing one’s closest parent at least weekly is much more common for those living within 1 h of them. Importantly for identifying an impact of ‘seeing weekly’ separately from proximity, 23% of persons who live within 1 h of their parents do *not* see their parents weekly, and 4.6% of those who live more than 1 h away do see them weekly. We have focused on frequency of physical contact because it is affected most by proximity to parents, but we also employed a measure of the frequency of contact by telephone, e-mail or letter (‘other contact’). Daily other contact declines with distance from parents, but weekly other contact does not vary a great deal with distance (result not shown).

**Table 5:** Distribution of frequency of seeing closest parent by proximity (column  %)

Frequency of seeing parent	Near	Far	All
Never	1.9	5.2	3.1
Less often	1.6	36.0	14.5
Several times per year	3.6	38.1	16.5
At least once per month	15.9	16.1	16.0
At least once per week	56.1	4.1	36.6
Daily	20.9	0.5	13.3
*N*	26,618	15,913	42,531
Row percentage	62.6	37.4	100

## Models for Long-Distance Movement

Our main probit regression model contains the family ties variables as well as the indicators of other local ties, variables related to the benefits of moving longer distances and the demographic control variables. Our estimates in Table 6 show average marginal effects calculated from the probit model and their standard errors.^5^ Most of the local ties effects on moving at least 40 km are all statistically significant and in the expected direction [Column (1)]. Although they may not at first sight appear large, they are when viewed relative to the annual mobility rate of 1.14%. In particular, the impact of having a parent living ‘far’ relative to ‘near’ is the same size as the mobility rate, and that of seeing a parent weekly is 41% of the mobility rate. One standard deviation more neighborhood interaction reduces long-distance mobility by 18% of the mobility rate.

**Table 6:** Average marginal effects from probit models of moving at least 40 km, any move, and OLS model of distance moved

	(1) Move 40 + km	(2) Move 40 + km	(3) Any move	(4) Distance moved^a^
Parent lives 1 h or more away	0.0114*** (0.0021)	0.0155*** (0.0017)	0.0164** (0.0052)	27.6*** (3.8)
See parent weekly	− 0.0047* (0.0019)	–	− 0.0068 (0.0047)	1.2 (3.0)
Dependent child	− 0.0019 (0.0013)	− 0.0027* (0.0013)	− 0.0093** (0.0035)	− 1.5 (2.9)
Neighborhood interaction	− 0.0021** (0.0007)	–	− 0.0144*** (0.0018)	− 1.3 (1.3)
Housing tenure				
Private tenant	0.0110*** (0.0023)	0.0110*** (0.0023)	0.0832*** (0.0052)	− 1.3 (3.5)
Homeowner	− 0.0001 (0.0023)	− 0.0006 (0.0023)	− 0.0283*** (0.0050)	3.9 (3.7)
Has partner	− .0067*** (0.0015)	− .0066*** (0.0015)	− 0.0200*** (0.0038)	− 3.9 (2.8)
Years in current residence	− 0.0001 (0.0002)	− 0.0001 (0.0002)	− 0.0036*** (0.0006)	0.6^†^ (0.3)
Education				
Post-compulsory	0.0034* (0.0015)	0.0035* (0.0014)	0.0017 (0.0040)	1.5 (2.7)
Degree or higher	0.0082*** (0.0017)	0.0086*** (0.0017)	0.0108* (0.0045)	14.6*** (3.7)
Rural	0.0040* (0.0016)	0.0033* (0.0016)	− 0.0030 (0.0043)	15.7*** (4.7)
Age (years)	− 0.0004*** (0.0001)	− 0.0004*** (0.0001)	− 0.0023*** (0.0002)	0.1 (0.2)
Female	0.0006 (0.0013)	0.0001 (0.0014)	0.0003 (0.0034)	− 0.6 (2.8)
Not UK born	0.0062*** (0.0018)	0.0064*** (0.0018)	0.0192*** (0.0047)	− 11.2** (4.1)
*N*	27,043	27,043	27,043	2484
Annual mobility rate	0.0114	0.0114	0.0919	–
*Pseudo R* ^2^	0.1468	0.1418	0.1546	*R*^2^ = 0.0615
Log-likelihood	− 1441.073	− 1449.481	− 7014.244	RMSE = 67.0

***p < 0.001; **p < 0.01; *p < 0.05; ^†^*p *< 0.10 (two-tailed test)

Descriptive statistics for the estimation sample are shown in Table 1

^a^Conditional on changing residence

Column (1) of Table 6 also indicates that having a dependent child does not have a significant impact on moving at least 40 km. This is also the case when using 30 and 50 km as the cutoff for a ‘distant move,’ but not when using 10 and 20 km as the cutoff, for which a dependent child decreases mobility. In contrast to what Table 4 suggested earlier, we found no evidence of a significant interaction effect between seeing parents weekly and the presence of a dependent child (results not shown). Furthermore, in line with our expectations, moving at least 40 km is more likely for (1) younger people, (2) those without a live-in partner, (3) private tenants (cf. homeowners or social tenants), (4) rural residents, (5) better educated people and (6) those not born in the UK.

When we added a variable indicating ‘daily other contact’ to our model for moving at least 40 km, its impact was virtually zero, and it left the other parameter estimates virtually unchanged. Thus, such frequent other contact is not an additional indicator of ties to parents which limit long-distance mobility over and above seeing them at least weekly.

Model (2) of Table 6, which omits both the parental contact and neighborhood interaction variables, produces an average marginal effect of parent living ‘far’ that is 36% higher than the effect in Model (1), indicating that the proximity to parents variable is partly a proxy for these elements of local ties.

Column (3) of Table 6 shows the results for moves of any distance. Although the average marginal effect of a parent living ‘far’ is larger in magnitude in the model for any move than in the model for moving at least 40 km, it is only 18% of the annual proportion making any move. Similarly, the seeing parent weekly and neighbor interaction effects in column (3) are 7% (compared with 41% for moves of 40 + km) and 23% of the mobility rate (compared with 18%).

There is concern that people who live over an hour from their parents are just more mobile people, and that is one of the reasons that they live far away, leading to a positive correlation between moving and having one’s closer parent living ‘far’ away. We have tried to address that by controlling for length of residence at the current address, which would be longer for less mobile people, but we have not found that it affects longer distance mobility. This lack of association of length of residence with movement was also found for distant moves using ‘distant’ cutoffs of 20 km and above (not shown). In the model of moves of any distance, however, the probability of moving is found to decline with time in the residence, leading to a significantly negative average marginal effect. One interpretation is that the deterrent effect on longer distance mobility from local ties, which is often captured as a negative impact of length of residence on mobility, is well represented by the parent proximity, parent contact and neighborhood interaction variables. Another interpretation is that our length of residence measure (in the dwelling) is less appropriate for longer distance moves because it does not capture the more salient residence in the community. In either case, longer time in residence is primarily associated with a lower probability of short-distance moves, in part capturing heterogeneity in mobility propensities.

Another approach to addressing heterogeneity in mobility propensities is to focus on currently mobile people and see how the parental and neighborhood tie variables affect the distance they move. The estimates of Model (4) of Table 6 indicate that conditional on moving, people living far from their parents move on average about 28 km farther than those who live near their parents. In contrast, weekly contact with parents and neighborhood ties have little impact on the distance moved.

Model (1) is our baseline model for other experimentation to test the robustness of our results. Table 7 indicates that the main results concerning the impact of family and neighborhood ties are not sensitive to the cutoff chosen for ‘distant move,’ although the impact of a parent living ‘far’ away relative to the mobility rate at that distance increases with the cutoff distance, as we would expect if moving longer distances is more likely to loosen family ties.

**Table 7:** Average marginal effects from probit models of moving: different cutoff distances

	(1) Move 50 + km	(2) Move 40 + km	(3) Move 30 + km	(4) Move 20 + km	(5) Move 10 + km
Parent lives 1 h or more away	0.0122*** (0.0022)	0.0114*** (0.0021)	0.0126**** (0.0024)	0.0135*** (0.0026)	0.0147*** (0.0030)
See parent weekly	− 0.0029 (0.0019)	− 0.0047* (0.0019)	− 0.0044* (0.0021)	− 0.0046* (0.0023)	− 0.0033 (0.0027)
Neighborhood interaction	− 0.0019** (0.0007)	− 0.0021** (0.0007)	− 0.0029*** (0.0008)	− 0.0034*** (0.0008)	− 0.0063*** (0.0010)
Number of moves	286	309	363	432	615
Mobility rate	0.0106	0.0114	0.0134	0.0160	0.0227
*Effect size relative to* (=* divided by*) *annual mobility rate*					
Parent lives 1 h or more away	1.15	1.00	0.94	0.84	0.65
See parent weekly	− 0.27	− 0.41	− 0.33	− 0.29	− 0.15
Neighborhood interaction	− 0.17	− 0.18	− 0.21	− 0.21	− 0.27

*N *= 27,043

***p < 0.001; **p < 0.01; *p < 0.05; ^†^*p *< 0.10 (two-tailed test)

The models also contain the other variables included in the models of Table 6

## Endogeneity?

Can we interpret the impact of proximity and/or frequency of contact as an ‘effect’ of local family ties? Even after controlling for length of residence and for movement in the current year (Model (4) in Table 6), there may still be concern that the impact of proximity represents heterogeneity in longer distance mobility propensities. What about contact with family and neighbors? Do frequent contact people have strong family ties, and as a consequence, both see their parent(s) often and have a low propensity to move longer distances? Or does exposure to frequent parental contact reduce their odds of moving longer distances? Similarly, do those who have stronger interactions with neighbors have a low inclination to move in any case and therefore make greater effort to know their neighbors? Or do ties with neighbors discourage movement over longer distances? These questions are difficult to answer.

To test further the robustness of the results for moves of at least 40 km, we estimated the model with different subsamples with an aim of reducing the heterogeneity in mobility propensities.^6^ First, we confined the analysis to people whose length of residence in any given year (up to the year preceding the potential move year) is 5 years or less (we do not know how *far* this ‘mobile group’ moved in the past 5 years). Column (2) of Table 8 shows the estimates of the impacts of the local ties variables in this sample. Among this mobile group, there are similar estimated average marginal effects on the probability of moving at least 40 km to those in the full sample [column (1)] for proximity to the close parent, weekly contact with parents and with interaction with neighbors, although not estimated as precisely.

**Table 8:** Average marginal effects of local ties variables, Model (2) of Table 6, different samples

	(1) Baseline, entire sample	(2) Length of residence ≤ 5 years	(3) Degree-educated	(4) Live ‘near’ parents
Parent lives 1 h or more away	0.0119*** (0.0020)	0.0191*** (0.0037)	0.0174*** (0.0039)	–
See parent weekly	− 0.0048** (0.0017)	− 0.0087* (0.0035)	− 0.0077^†^ (0.0044)	− 0.0033* (0.0013)
Neighborhood interaction	− 0.0017** (0.0006)	− 0.0022^†^ (0.0012)	− 0.0017 (0.0012)	− 0.0008 (0.0005)
N	31,382	11,781	9820	20,372
Annual mobility rate	0.0112	0.0185	0.0202	0.0042
*Pseudo R* ^2^	0.1563	0.1323	0.1591	0.0790
*Effect size relative to* (=* divided by*) *annual mobility rate*				
Parent lives 1 h or more away	1.06	1.03	0.86	–
See parent weekly	− 0.43	− 0.47	− 0.38	− 0.79
Neighborhood interaction	− 0.15	− 0.12	− 0.08	− 0.19

***p < 0.001; **p < 0.01; *p < 0.05; ^†^*p *< 0.10 (two-tailed test)

Models contain the other variables in Model (2) of Table 6, except length of residence

Another way to reduce long-distance heterogeneity in mobility is to focus on a group who we know are more mobile over longer distances: people who have a university degree. In Column (3) of Table 8, we see a similar strong positive association between moving at least 40 km and living far from parents and a negative association between mobility and seeing parents weekly.

Next, we focused on estimating the impacts of frequency of contact among those living near to parents. The sample was therefore confined to people living within 1 h of their parents (65% of the sample). Among this group, who tend to be less mobile over longer distances, seeing parents at least weekly significantly reduces the odds of moving at least 40 km (Column (4) of Table 8), a large effect relative to the low average probability of moving that distance for this group.

Another approach is to only use ‘within-person’ variation; that is, everyone has their own underlying mobility propensity and we compare the local ties’ variables between situations when they move at least 40 km and when they do not move that distance. In effect, each person is their own control. We investigated this with a conditional logit model, which only uses persons observed moving at least 40 km in one wave but not the other (recall we have only two pairs of waves in which we have the local ties measures). Only neighborhood interactions approach statistical significance (*p* value = 0.11), and they have a similar relative impact as in the broader sample. The insignificance of the parental proximity/contact in these regressions might reflect insufficient changes in parental proximity/contact status between the two waves relative to the size of the sample. By dropping the parental proximity and contact variables, we obtained more precise estimates of the impact of neighborhood interactions on moving at least 40 km: a logit coefficient (SE) of − 0.160 (0.074). That is, in years in which people had less interaction with neighbors for whatever reason (perhaps a close neighbor moved away), they are more likely to move 40 km or farther in the subsequent year. Of course, the conditional logit sample overrepresents frequent long-distance movers, who are a highly select category.

None of these statistical exercises is a solution to the endogeneity issues, and we cannot rule out that people who move long distances are characterized by relevant unobserved traits which are correlated with distance from parents, frequency of contact and interactions with neighbors. But comparing estimates from the different definitions of a ‘distant move’ and different samples (Tables 7 and 8), there are three strong consistencies in associations with mobility of at least 40 km: Living near parents reduces it, as does seeing parents weekly, and more interactions with neighbors usually reduce it. Furthermore, the ‘effects’ of these parent and neighborhood ties are large relative to the underlying distant mobility rate, particularly close proximity to parents.

## Conclusions

We investigated the association between living far from versus close to parents and the likelihood of migration in Britain. As hypothesized and as in previous research (e.g., by Mulder and Malmberg 2014 for Sweden), we found a strong association between these two. New findings compared with previous research are that this association is much weaker when we take into account that those who had frequent contact with parents or more interaction with neighbors are much less likely to make long-distance moves. But the association does not disappear. Also new were various attempts to address endogeneity issues.

The usual thought experiment in interpreting causal effects is random assignment of location relative to parents (‘less than 1 h’ compared with ‘1 h or more’) or frequency of contact with them (at least weekly or not). If our results are causal, a move far away from parents which is generated for reasons unrelated to family ties (e.g., for higher education or a first job) would then increase longer distance mobility in subsequent years relative to those who stay closer to their parents and see them often.

Another interpretation of our results is that the impact of frequent contact/close proximity reflects strong family ties developed over a person’s life. It is an attribute of that person that constrains his or her movement over longer distances, and differs from, for example, the impact of local friendship networks, or neighborhood interaction, on longer-distance movement (Belot and Ermisch 2009), which are local ties that are developed by residing in a particular area. In contrast, family is given and the ties are developed over a much longer period than current length of residence. Our results indicate that if a person lives near their parents, then there is strong persistence in remaining nearby, which affects migration for labor market reasons.

Stronger local ties with parents and neighbors doubtless bring many benefits to the people involved, but our results indicate that the trade-off is that those with stronger ties are less mobile over longer distances.

## Appendix on Attrition

Define the probability $P[M_{i} = j]$, where *j *= 1 if the person moves at least 40 km between waves of the panel and *j *= 0 if they do not, and the probability $P[R_{i} = k],$ where *k *= 1 if the person remains in the panel between consecutive waves and *k *= 0 if they do not. In the analysis in the paper, we have estimated how variables affect the probability of moving at least 40 km *conditional* on remaining in the sample, $P[M_{i} = 1 |R_{i} = 1 ]$, because we do not know whether people moved and how far if they drop out of the panel.^7^ We would have liked to estimate how the variables affected the unconditional probability of moving, $P[M_{i} = 1]$.

By Bayes Theorem,

$$P[M_{i} = 1 |R_{i} = 1 ]= \frac{{P[R_{i} = 1 |M_{i} = 1 ]\cdot P\left[ {M_{i} = 1} \right]}}{{P\left[ {R_{i} = 1} \right]}}$$

Re-arranging terms, we obtain:

$$P[M_{i} = 1] = \frac{{P[M_{i} = 1 |R_{i} = 1 ]\cdot P\left[ {R_{i} = 1} \right]}}{{P[R_{i} = 1|M_{i} = 1]}}$$

Define $q_{i} = \frac{{P[R_{i} = 1|M_{i} = 0]}}{{P[R_{i} = 1|M_{i} = 1]}}$, and note that

$$P[R_{i} = 1] = P\left[ {R_{i} = 1 |M_{i} = 1} \right] \cdot P[M_{i} = 1\left] { + P\left[ {R_{i} = 1 |M_{i} = 0} \right] \cdot (1 - P[M_{i} = 1} \right]).$$

If remaining in the panel to the subsequent wave is independent of long-distance mobility, then $q_{i}$ = 1 and the conditional and unconditional probabilities of long-distance mobility are the same. It is, however, plausible that $q_{i}$ > 1 because it is more difficult to follow people in the panel when they move long distances.^8^

From these relationships, one can derive:

$$P[M_{i} = 1] = \frac{{P[M_{i} = 1 |R_{i} = 1 ]q_{i} }}{{1 - P[M_{i} = 1 |R_{i} = 1 ]\left( {1 - q_{i} } \right)}}$$

We obtain from this equation the derivative

$$\frac{{dP[M_{i} = 1]}}{{dP[M_{i} = 1 |R_{i} = 1 ]}} = \frac{{q_{i} }}{{\left[ {1 - P[M_{i} = 1 |R_{i} = 1 ]\left( {1 - q_{i} } \right)} \right]^{2} }}$$

Given the conditional mobility probabilities we observe, the denominator of the derivative is very close to unity for plausible values of $q_{i}$. From Table 6, the average $P[M_{i} = 1 |R_{i} = 1 ]= 0.0112$. Thus, even, for example, if $q_{i}$ = 1.2, the denominator is 1.0045. The effect of any variable, such as living ‘far’ from parents, on $P[M_{i} = 1]$ is therefore greater by a factor of $q_{i}$ than its effect on $P[M_{i} = 1 |R_{i} = 1 ]$, the latter of which we estimate in the paper makes the estimates shown in the paper conservative ones. This is consistent with the simulation study in Washbrook et al. (2014; Table 7).

A variable of interest could, of course, also affect $$q_{i}$$; i.e. retention in the panel may change more or less among long-distance movers compared with non-movers or short-distance movers. We cannot observe $$q_{i}$$ because we do not observe mobility for those who leave the panel, but only observe how variables in our models affect retention in the panel between waves, $$P[R_{i} = 1]$$. Also, $$q_{i}$$ varies among people for reasons we cannot observe. Washbrook et al. (2014) develop one model in which retention depends causally on mobility (directly affecting $$q_{i} )$$ and another in which the association between retention and mobility is assumed to be due to omitted variables that are associated with both processes. In their analysis, the results concerning effects of covariates on mobility are not sensitive to which model is used to construct the likelihood.

Appendix Table 9 shows the effects of our key family tie and neighborhood variables on $P[R_{i} = 1]$. For instance, living far from parents reduces the probability of remaining in the sample, but this could come about because it increases long-distance mobility without affecting the components of $q_{i}$. In the case of seeing parents weekly, it does not appear to affect $P[R_{i} = 1]$, despite the fact that it does reduce $P[M_{i} = 1 |R_{i} = 1 ]$, suggesting some compensating changes in the components of $q_{i}$. Finally, more neighborhood interaction increases the probability of retention, but again this could be because it reduces long-distance mobility. The effects are small relative to the overall probability of retention (0.80) or dropout (0.20).

**Table 9:** Average marginal effects of local ties variables on retention in the panel

	(1)^a^
Parent lives 1 h or more away	− 0.014* (0.006)
See parent weekly	− 0.002 (0.005)
Neighborhood interaction	0.007*** (0.002)
N	45,575
Annual retention rate	0.802
*Pseudo R* ^2^	0.024

^a^Models contain the other variables in Model (2) of Table 6, except length of residence

****p *< 0.001; ***p *< 0.01; **p *< 0.05; ^†^*p *< 0.10 (two-tailed test)

The analysis above suggests that the estimates of the magnitude of the effects in the paper are likely to understate the true effects on long-distance mobility, but the understatement may be small if the panel retention rate of non-movers is not too much larger than among movers (i.e. $q_{i}$ is relatively small).

## Footnotes

1 This approach to developing a score from the five statements is similar to principal component analysis, and indeed the correlation between the MCA score and the principal component (PC) analysis score is 0.976. In fact, if we just summed the scores from the five questions, giving 0 to strongly disagree and 5 to strongly agree, then the correlation of that sum with the PC score is 0.998 and with the MCA score is 0.974. Furthermore, if we substitute either the sum or the PC score for the MCA score in the models below, the marginal effects remain virtually unchanged. So it appears to make little difference how we summarize these neighborhood data.

2 This is derived from the Office for National Statistics Rural and Urban Classification of Output Areas.

3 Because the parents are older than the respondents, their likelihood of moving is considerably smaller than the respondents’ (e.g., Bernard et al. 2014).

4 We attempted analysis of the impact of covariates on the probability of moving closer, but with only 135 observations, it is difficult to achieve any precision in estimation. At best there is some hint that older people are less likely to move closer to their parents: For each year older, the average marginal effect on the probability of moving closer is − 0.0047 (SE = 0.0035).

5 Age of child mattered for the probability of any move, but for purposes of comparison across columns we have also used the simple presence of dependent child indicator in model (3) of Table 6. For information, having a child aged 0–2 encourages a residential move, but having a child aged 5–5 discourages a move. The significant negative impact of the presence of dependent child in column (3) of Table 6 reflects the effects for older children.

6 In these regressions, length of residence was omitted from the regressions because of its statistical insignificance and the fact that we lose 43 moves of at least 40 km and 4339 person-years because there are missing data on length of residence.
